# Designing and screening of universal drug from neem (*Azadirachta indica*) and standard drug chemicals against influenza virus nucleoprotein

**DOI:** 10.1186/s12906-016-1469-2

**Published:** 2016-12-16

**Authors:** Aftab Ahmad, Muhammad Rizwan Javed, Abdul Qayyum Rao, Tayyab Husnain

**Affiliations:** 1 Center of Excellence in Molecular Biology (CEMB), University of the Punjab, West Canal Road, 53700 Lahore, Pakistan; 2 Department of Bioinformatics and Biotechnology, Government College University Faisalabad (GCUF), Allama Iqbal Road, 38000 Faisalabad, Pakistan

**Keywords:** Influenza virus, Nucleoprotein, Neem leaf extract, Molecular docking, Universal drug

## Abstract

**Background:**

Different strains of influenza virus are affecting a large number of people worldwide. Many synthetic antiviral medicines are available for influenza virus in the market. But still there is a need for the development of universal drugs against these strains of influenza virus.

**Methods:**

For this purpose conserved residues within the influenza virus nucleoprotein have been retrieved. The drugs, previously known to have antiviral properties, were screened to identify the best candidate universal drug against Influenza virus strains. Compounds from leaf extracts of neem, were also screened to identify the natural drugs without side effects.

**Result:**

Molecular docking identified three potential compounds (Nimbaflavone, Rutin, and Hyperoside) having perfect binding with reported conserved residues (ASP302, SER50) of influenza virus nucleoprotein that is involved in the binding of drugs. Further analysis showed Hyperoside as a universal drug against various influenza strains. Some chemical drugs were also evaluated through screening against nucleoprotein. The results showed six drugs (OMS, CBX, LGH, Naproxen, BMS-883559, and BMS-885838) which were interacting with same conserved residues (ASP302, TYR52, SER50, GLY288, SER376, and ARG99) as were found in the case of neem phytochemicals. Hyperoside from neem leaf extract along with drugs LGH, Naproxen, BMS-885838, and BMS-883559 showed best interactions with conserved residues of nucleoprotein.

**Conclusion:**

The compound Hyperoside from neem leaf extract along with drugs LGH, Naproxen, BMS-885838, and BMS-883559 showed best interactions with conserved residues of nucleoprotein. So these compounds have been identified for their potential against influenza strains to be utilized as a universal drug.

## Background

Influenza is a significant health problem due to its rapid transmission and high mortality rate. It is a respiratory infection caused by the influenza virus [1] belonging to the family Orthomyxoviridae. The virus has single stranded and segmented RNA genome which encodes 8 proteins. For the propagation of influenza virus, the nucleoprotein (NP) matrix (M1, M2), neuraminidase (NA), hemagglutinin (HA) and three viral polymerase subunits (PB1, PB2 and PA) play an active role [2]. Influenza virus is classified into three groups and has more than 10 strains. Its genome is highly variable having high rate of mutation which make it resistant to many drugs.

Previously the disease was cured by using nucleic acid protein inhibitors, neuraminidase inhibitors (e.g. Zanamivir, Oseltamivir), ion channel blockers (e.g. Amantadine, Rimantadine) and siRNA technique [3]. The whole concept of the gene regulation and proteome function can be used to predict projected motifs antiviral drug target of influenza A virus [4]. Host specific epitopes of influenza A virus surface proteins NA and HA, have been predicted which could support in designing the drugs against Influenza virus [5]. Oseltamivir and Zanamivir have been proved as efficient inhibitors of NA. Due to the mutation in the active site of NA and HA, resistance of influenza virus against these drugs has been reported [6].

Nucleoprotein (NP) is a well-studied highly conserved trimeric protein, having a main role in the influenza virus life cycle. It acts as an RNA single stranded binding protein. NP is a structural protein of ribonucleoprotein particles (RNPs). It has a great role in RNPs trafficking between the nucleus and cytoplasm. NP is also involved in the replication of RNPs. NP directly binds with M1, PB1 and PB2. In infection stage, RNPs are being released in the cytoplasm. Due to the interaction of host proteins with NP the process of RNPs import in nucleus and export in the cytoplasm takes place. The sequence of NP only differs 11% in all reported strains, contributing towards its suitability as a target for universal drug development against all influenza virus strains [7, 8].

By random screening, Nucleozin is recently discovered compound, which induces NP aggregation, by inhibiting viral replication and accumulation of NP in the nucleus. Successive structure determination of NP bound with an analog of Nucleozin identified a NP dimer with back-to-back organization between the monomers, causing an interface which docks, two anti-parallel ligand molecules around the two ligand binding sites. Mutations causing resistance against Nucleozin have also been reported [8, 9].

Medicinal plants are very important for treatment of different diseases, mainly in the countries where there are insufficient resources. Uses of traditional medicines are mainly encouraged in most of the world population [10]. These traditional medicines have least side effects than other allopathic medicines, one of the major reasons to isolate and process these compounds from plants [11]. Neem is a medicinal plant and has been grown for its universal importance in recent years. Neem has been extensively used in Ayurveda, Unani, and Homeopathic medicines. It has a huge range of chemically and structurally different biologically active chemicals. More than 140 chemically active compounds have been isolated from different parts of this plant including i.e. flowers, leaves, seeds, roots, fruits, and bark and are being used traditionally as a cure for many diseases. These active compounds have been identified as anti-inflammatory, anti-ulcer, anti-hyperglycaemic, immune-modulator, anti-mutagenic, anti-carcinogenic, anti-oxidant, and anti-viral drugs [12].

Neem elements are mainly divided in two groups: Non-isoprenoids and Isoprenoids. The non-isoprenoids comprise of proteins, sulphurous compounds, carbohydrates and polyphenolics including dihydrochalcone, flavonoids, coumarin, and aliphatic compounds. The isoprenoids consist of di-terpenoids and tri-terpenoids which include azadirone, protomeliacins, limonoids and some derivatives such as nimbin, vilasinin, salanin and azadirachtin [10]. By an alcoholic extract of neem leaves a dose dependent substantial decrease in blood pressure has also been reported [13].

In the background of above debated medications by neem, this study has been designed to screen active compounds of neem against the influenza virus nucleoprotein through molecular docking and to study their interaction pattern. Moreover, already reported ten drugs against various influenza virus strain nucleoproteins were also used for comparison through molecular docking and determination of their interaction patterns with NP conserved residues.

## Methods

*In-silico* analysis of neem leave’s active chemicals against influenza virus nucleoprotein was carried out. To acquire this, chemical structures of compounds of neem leaves were retrieved in MOL format from chemical database PubChem available on NCBI website. Some of the chemical compounds were drawn in MOL format by using Chemdraw software. Nucleoprotein structure [PDBID: 3RO5] used for docking purpose was downloaded from Protein Data Bank (PDB). For molecular docking analysis, Molecular Operating Environment (MOE) software was used [14].

### Finding the nucleoprotein conserved residues within and among the influenza strains

**Fig. 1:**
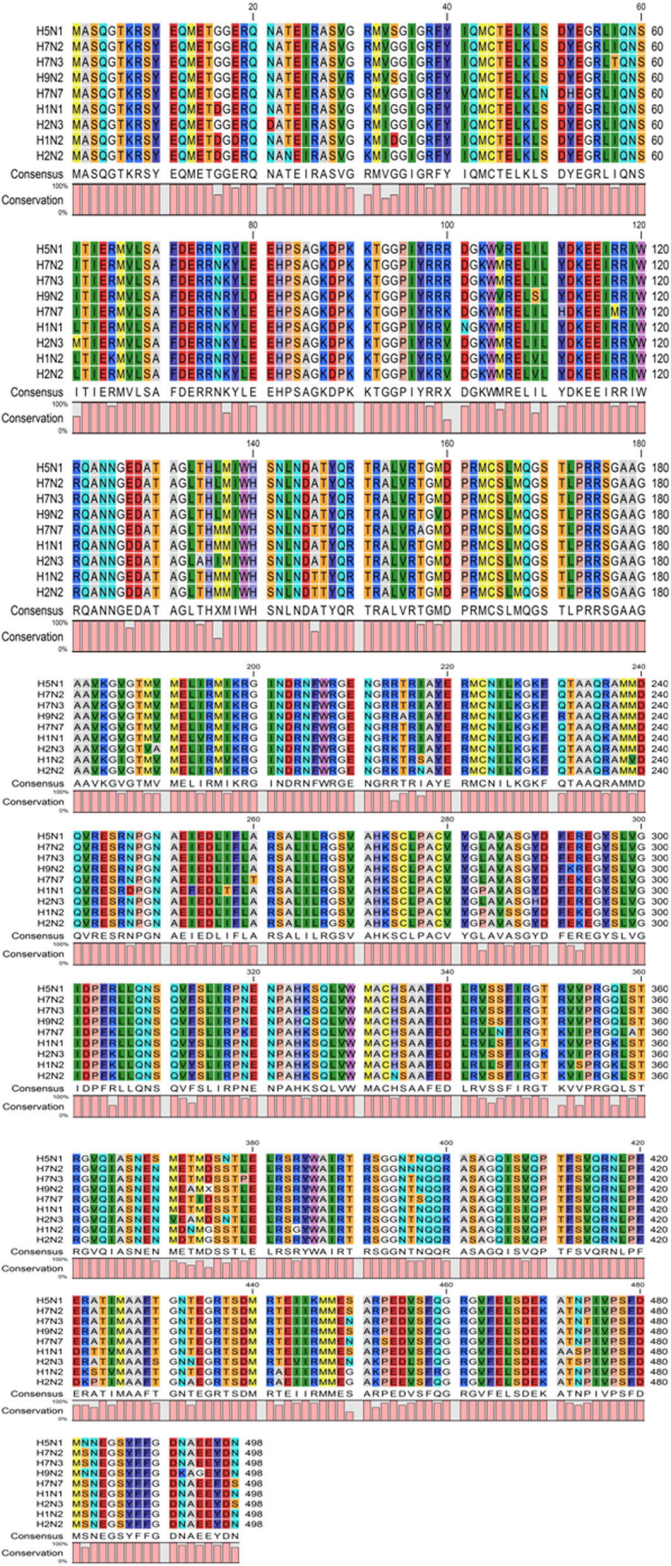
Multiple sequence alignment of influenza virus nucleoprotein consensus sequences of each strain (i.e. H5N1, H7N2, H7N3, H9N2, H7N7, H1N1, H2N3, H1N2 and H2N2) using CLC Genomics Workbench 8. For the development of each consensus sequence, all the available nucleoprotein sequences of above said strains were retrieved from NCBI database and were converted to consensus sequences using CLC Genomics Workbench 8. The colored bars at the bottom are representing the conservation %age

In order to find the conserved residues of nucleoprotein within and among the strains of influenza virus active against human (H1N1, H1N2, H2N2, H2N3, H5N1, H7N2, H7N3, H7N7 and H9N2), the available nucleoprotein sequences of each strain were retrieved from NCBI protein database. At first, the retrieved nucleoprotein sequences of each strain were aligned by multiple alignment tool Clustal Omega (http://www.ebi.ac.uk/Tools/msa/clustalo/) to develop a consensus sequence for each strain and to identify conserved residues within the strain. The conserved residues consensus sequences of all the strains were again aligned using the CLC Genomics Workbench 8 to get the final conserved consensus sequence among the all strains (Fig. 1).

### Molecular docking

#### Preparation of protein structure (Receptor)

Three-dimensional model of influenza virus Nucleoprotein was retrieved from PDB [PDB ID: 3RO5]. All the water molecules were removed by using MOE software. After removal of water molecules, hydrogen atoms were added to the receptor protein. Optimization of receptor molecule was achieved through energy minimization and 3D protonation by using AMBER99 force field option of MOE. The gradient was 0.05 and receptor was minimized unless root mean square gradient reached below 0.05. After that the receptor protein was 3D protonated and then hydrogen molecules were hidden by using hide molecule option of MOE.

There were three ligand molecules attached to the receptor molecule because nucleoprotein is a trimeric protein, so one ligand was attached to one chain of protein. Two of them were deleted by using sequence bar to get the single docking site and then pocket of the remaining ligand was used as the docking site. Surface and maps option of MOE was used to point out the surface of the docking site and pocket residues. This energy minimized and 3D protonated receptor molecules were then used for docking analysis.

#### Preparation of ligand structure and construction of database

The structures of neem leave’s biologically active compounds and reported drugs against influenza virus nucleoprotein were downloaded from the PubChem database in 2D format. Some structures of chemical compounds were not presented in the PubChem database so their 2D structures were retrieved from literature and drawn in 3D format by using ChemDraw software. For preparation of ligand structures for docking, hydrogen atoms were added to each ligand and their energy was minimized by using the MMFF94X force field at 0.05 gradients. Then these ligand structures were saved in .mol2 file format.

The two databases (one for neem leaf chemicals and other for already reported drugs) of ligands were created separately in MOE software. Ten previously reported drugs included in the database were: Nucleozin; (+)-(*S*)-2-(6-methoxynaphthalen-2-yl)-propanoic acid [Naproxen]; 1H-1,2,3-triazole-4-carboxamide [CBX]; AGN-PC-09RM4KT; 4-(2-chloro-4-nitrophenyl)piperazin-1-yl][3-(2-chloropyridin-3-yl)-5-methyl-1,2-oxazol-4-yl]methanone [OMM]; 4-(5-bromanyl-3-methyl-pyridin-2-yl)piperazin-1-yl]-[3-(2-chlorophenyl)-5-methyl-1,2-oxazol-4-yl]methanone [OMS]; 4-(2-chloro-4-nitrophenyl)piperazin-1-yl][3-(2-methoxyphenyl)-5-methyl-1,2-oxazol-4-yl]methanone [LGH]; N-[4-chloranyl-5-[4-[[3-(2-methoxyphenyl)-5-methyl-1,2-oxazol-4-yl]carbonyl]piperazin-1-yl]-2-nitro-phenyl]furan-2-carboxamide [BMS-885986]; N-[4-chloranyl-5-[4-[[3-(2-methoxyphenyl)-5-methyl-1,2-oxazol-4-yl]carbonyl]piperazin-1-yl]-2-nitro-phenyl]pyridine-2-carboxamide [BMS-885838]; and N-[4-chloranyl-5-[4-[[3-(2-methoxyphenyl)-5-methyl-1,2-oxazol-4-yl]carbonyl]piperazin-1-yl]-2-nitro-phenyl]thiophene-2-carboxamide [BMS-883559] [7, 15, 16]. These databases were saved in .mdb format and were further used for docking against the target receptor protein.

#### Docking analyses

After preparation of receptor protein and ligand molecules, molecular docking was executed against the databases mentioned earlier. Ligands were docked with receptor by choosing the pocket of already present ligand in the receptor protein by using MOE. There were 20 amino acids in that pocket; LEU315, SER376, TRP104, LEU56, TYR313, GLU53, TYR52, SER50, ARG99, GLU294, ALA284, TYR289, ARG26, ASP302, ARG305, TYR296, ASP290, ALA284, SER314, and LYS288. Docking output database files containing receptor ligand complex were saved in .mdb format. The docked complexes were sorted with respect to increasing *S* value (the final score to indicate binding free energy). The complexes with minimum *S* values were taken to evaluate the interactions of ligands with the active site residues of the receptor protein. The best hydrogen bonding and π-π interactions were analyzed by the ligX option of MOE.

## Results

### Finding conserved residues within and among influenza virus nucleoprotein

To find the conserved residues in all the strains of influenza virus (Fig. 1), alignment was done by using Clustal Omega and CLC Genomics Workbench 8 as described earlier. In the Fig. 1 conservation of residues is shown by vertical bars. The alignment showed that nucleoprotein is a highly conserved protein (81.73%).

### Docking analyses against neem leaf chemicals

**Fig. 2:**
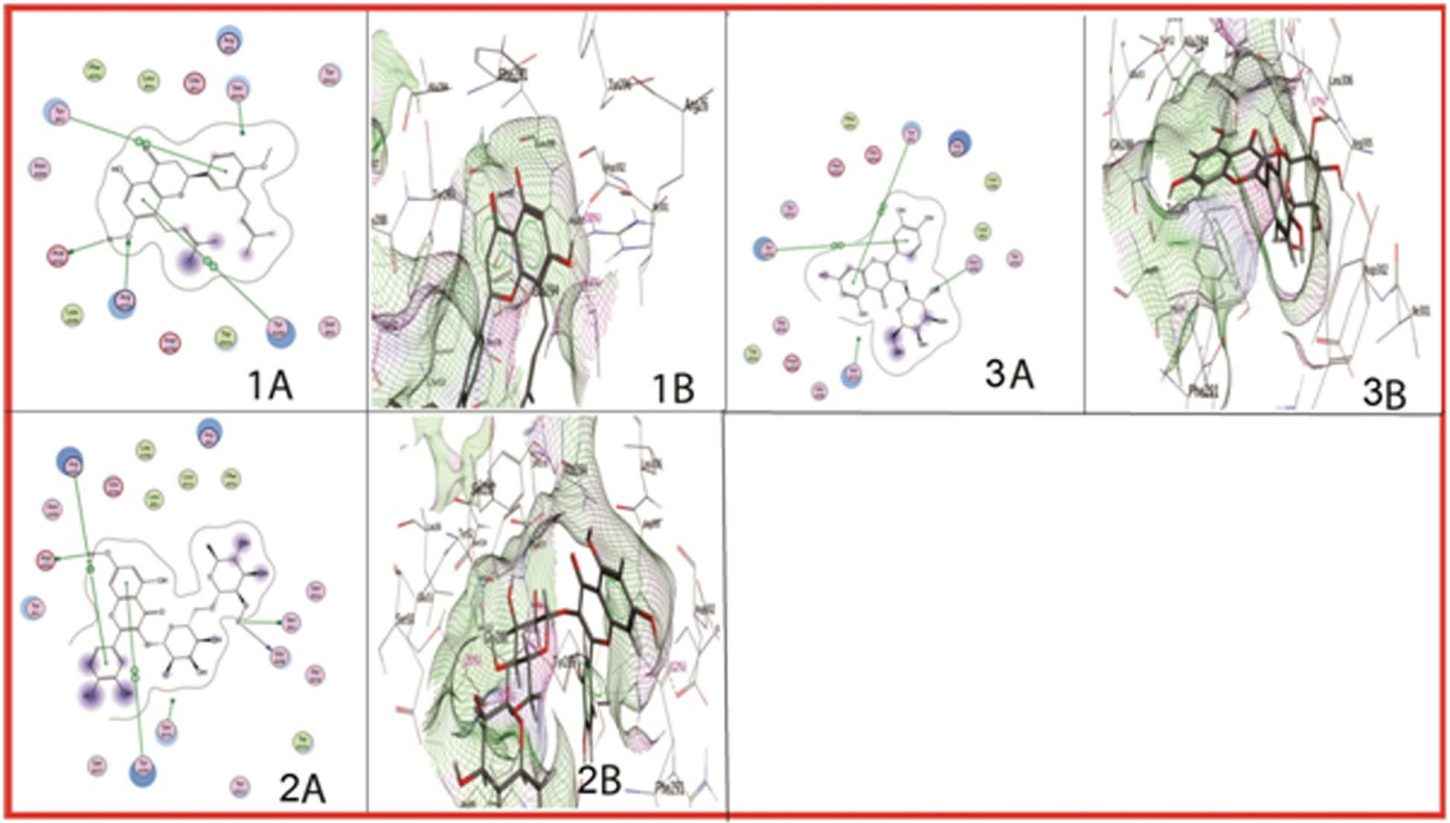
Interaction diagrams of phytochemicals from Neem leaf extract compounds with influenza virus nucleoprotein. Where; 1A & 1B are two dimensional and three dimensional interaction diagrams of ARG305, TYR289, TYR52 and ASP302 residues of influenza virus nucleoprotein with Nimbaflavone, respectively; 2A & 2B are showing the interaction of ARG305, TYR289, ASP302, SER50 and GLY288 residues of nucleoprotein with Rutin; and 3A & 3B are illustrating the interaction of ASN309, TYR289 and TYR52 residues with Hyperoside. Interaction diagrams were attained by using ligand interaction analysis feature of MOE

Docking of influenza virus nucleoprotein against neem leaf chemicals resulted in 3 complex conformations. Nimbaflavone showed least *S*-score and interacted with the ARG305, TYR289, TYR52, and ASP302 residues of nucleoprotein (Table 1; Fig. 2: 1A & 1B). The other two compounds (Rutin and Hyperoside) were also having a lower *S*-score and strong hydrogen bonds with multiple residues of the nucleoprotein (Table 1, Fig. 2: 2A-3B). All these compounds showed interactions with TYR289 residue of the selected pocket of nucleoprotein.

**Table 1:** Docking score (*S*) and interaction sites of neem phytochemicals against influenza virus nucleoprotein

Sr. no.	Compound name (Drug no.)	Score (*S*)	Nucleoprotein interacting residues
1	Nimbaflavone (14492795)	−32.0315	ARG305, TYR289, TYR52, ASP302
2	Rutin (5280805)	−31.2659	ARG305, TYR289, ASP302, SER50, GLY288
3	Hyperoside (5281643)	−28.3207	ASN309, TYR289, TYR52

ARG305 residue showed interaction with Nimbaflavone and Rutin, while TYR52 showed the interactions with Nimbaflavone and Hyperoside. ASP302 showed interaction with both Nimbaflavone and Rutin while SER50 as well as GLY288 interacted with Rutin only.

### Docking analysis against reported drugs

**Fig. 3:**
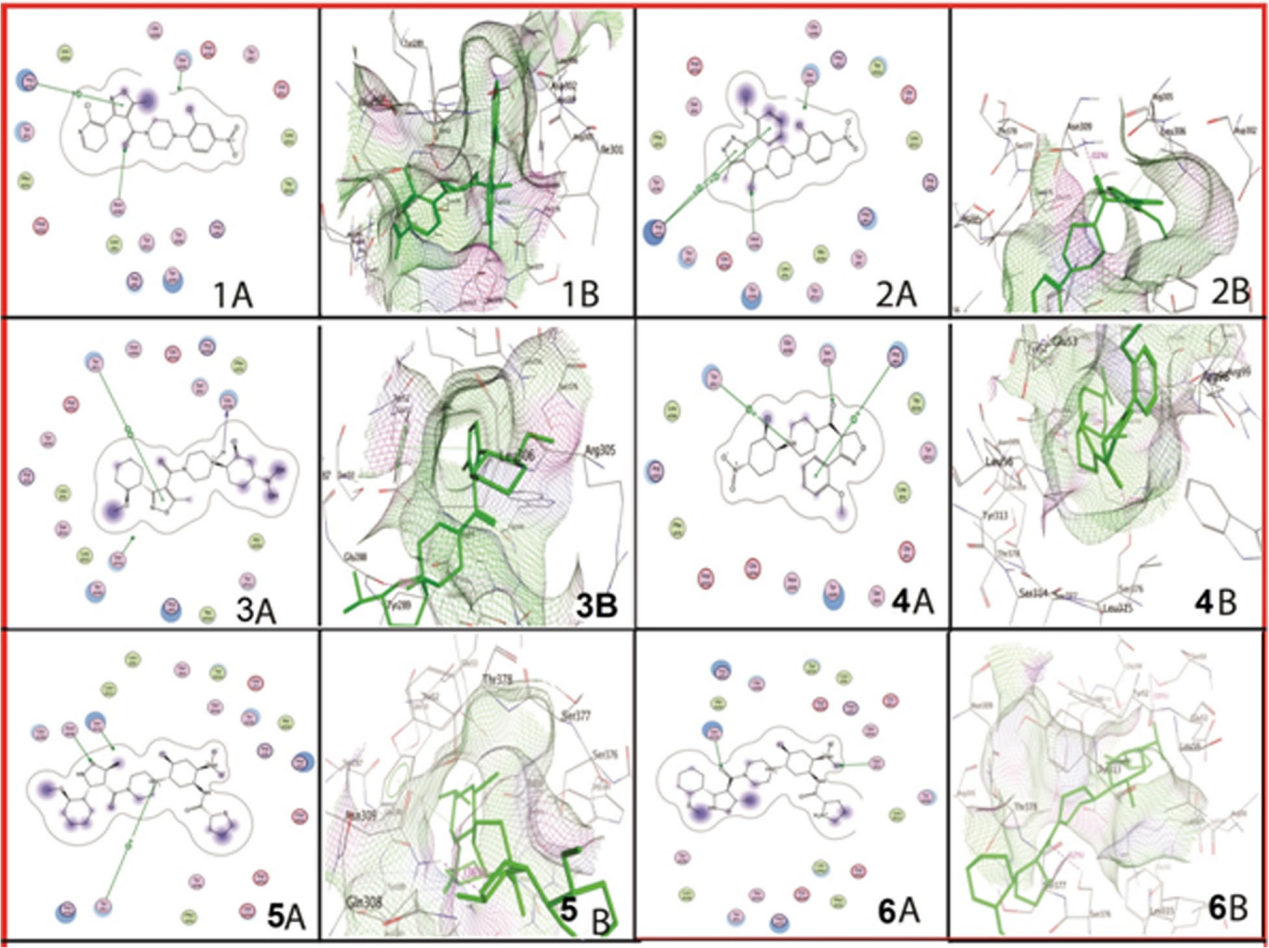
Interaction diagrams of reported drugs with influenza virus nucleoprotein. Whereas; 1A & 1B are showing binding of OMS with ARG305, ASN309; 2A & 2B are illustrating CBX interactions with ARG305, ASN309; 3A & 3B are showing interactions of LGH with TYR52, GLY288 residues of nucleoprotein, respectively. Furthermore, 4A & 4B are describing naproxen interactions with ARG99, TYR52, SER376 residues; While 5A & 5B of BMS-883559; 6A of BMS-885838; and 6B of BMS-885838, respectively. Interaction diagrams were attained by using ligand interaction analysis feature of MOE

Docking of influenza virus nucleoprotein against previously reported drugs resulted in six complex conformations (Table 2; Fig. 3). These drugs were also selected on the basis of low *S*-value and the interaction with the nucleoprotein residues. The interactions were shown in Fig. 3. The OMS, CBX and LGH showed interactions with (ARG305, ASN309), (ARG305, ASN309) and (TYR52, GLY288) respectively, while naproxen with ARG99, TYR52, SER376; BMS-883559 with ASN309, TYR52 and BMS-885838 with SER50, SER376, respectively.

**Table 2:** Docking score (*S*) and interaction sites of reported drugs against influenza virus nucleoprotein

Sr. no.	Compound name (Abbreviation or Drug No.)	Score (*S*)	Nucleoprotein interacting residues
1	[4-(5-bromanyl-3-methyl-pyridin-2-yl)piperazin- 1-yl]-[3-(2-chlorophenyl)-5-methyl-1,2-oxazol- 4-yl]methanone (OMS)	−24.9343	ARG305, ASN309
2	1H-1,2,3-triazole-4-carboxamide (CBX)	−24.0769	ARG305, ASN309
3	4-(2-chloro-4-nitrophenyl)piperazin-1-yl][3-(2-methoxyphenyl)-5-methyl-1,2-oxazol-4-yl]methanone (LGH)	−20.1668	TYR52, GLY288
4	(+)-(*S*)-2-(6-methoxynaphthalen-2-yl)-propanoic acid (Naproxen)	−16.7973	ARG99, TYR52, SER376
5	N-[4-chloranyl-5-[4-[[3-(2-methoxyphenyl)- 5-methyl-1,2-oxazol-4-yl]carbonyl]piperazin- 1-yl]-2-nitro-phenyl]thiophene-2-carboxamide (BMS-883559)	−16.0942	ASN309, TYR52
6	N-[4-chloranyl-5-[4-[[3-(2-methoxyphenyl)- 5-methyl-1,2-oxazol-4-yl]carbonyl]piperazin- 1-yl]-2-nitro-phenyl]pyridine-2-carboxamide (BMS-885838)	−8.5787	SER50, SER376

## Discussion

Nucleoprotein (NP) is reported to have a main role in the life cycle of influenza virus. It acts as a single stranded RNA binding protein that plays a significant role in ribonucleoprotein particles (RNPs) trafficking between the nucleus and cytoplasm. It is structural protein of RNPs that is involved in their replication. Consensus sequences taken from aligning all the sequences of each influenza strains, separately, were used for the further alignment. The results of the consensus sequence’s further alignment showed that NP is near about 81.78% conserved in all the influenza strains. These results were in accordance with Portela and Digard [8], who reported that NP only differs 11% in all reported strains. These reasons are contributing towards its suitability as a target for universal drug development against all influenza virus strains [7, 8].

For docking purpose the receptor and ligands were prepared and docked as described by Ahad et al., [17]. The docking showed that ARG305, TYR289, TYR52, ASP302, SER50, GLY288, and ASN309 were the residues involved in the interaction with the neem leaf extract chemicals (Table 1). These results are in accordance with the findings of Cianci et al., [18] who also reported the interaction of Aryl piperazine amides inhibitor with TYR52, TYR289, and ASN309 pocket residues. ASP302, GLY288 and ASN309 were found to be conserved residues in the nucleoprotein of influenza virus strains. However in H7N7 influenza strain, TYR50 and TYR52 were substituted with ASN50 and HIS52, respectively, while in H2N3 strain TYR289 was amended with HIS289. ARG305 was common among H5N1, H7N2, H7N3, H9N2 and H1N1 strains, but substituted with LYS305 in H7N7, H2N3, H1N2 and H2N2 strains (Fig. 1).

Out of investigated neem leaf chemicals, Hyperoside have not shown any interaction with ARG305 as compared to other two drugs. Furthermore, TYR289 and TYR52 interacting residues were not present in H2N3 and H7N7, respectively. These observations suggested that Hyperoside can be used as antiviral drug against entire influenza virus strains except H2N3 and H7N7. Literature also depicts that Hyperoside and Nimbaflavone have antiviral activities, while Rutin has antiviral, antifungal and antimicrobial activities [19]. Hyperoside is also reported to be considerably effective against hepatitis B virus (HBV) [20, 21].

The previously reported drugs OMS, CBX and LGH showed interactions with (ARG305, ASN309), (ARG305, ASN309) and (TYR52, GLY288), respectively, while naproxen showed with ARG99, TYR52, SER376; BMS-883559 with ASN309, TYR52 and BMS-885838 with SER50, SER376, respectively. These results were also in accordance with Lejal, et al., [22], who have reported the interaction of naproxen with NP of influenza virus.

The docking of nucleoprotein with already reported drugs has shown that SER50, TYR52, ARG99, GLY288, ARG305, ASN309, and SER376 residues of nucleoprotein were involved in interaction. Out of these, ARG99, GLY288, ASN309, and SER376 residues were found to be conserved among influenza virus strains (Fig. 1). SER50 and TYR52 were changed in H7N7 strain with ASN50 and HIS52, respectively. TYR289 has been substituted with HIS289 in H2N3 influenza strain. ARG305 was found to be common among H5N1, H7N2, H7N3, H9N2, and H1N1 strains, but substituted with LYS305 in H7N7, H2N3, H1N2 and H2N2 strains as mentioned before. So from Table 2, it is concluded that drugs LGH, Naproxen, BMS-885838 and BMS-883559 interacted with conserved residues, so they can be used as universal drugs against influenza virus strains except H7N7 because one of its interacting residue is missing. This evaluation corresponds with Davis et al., [23] who proposed that 289 and 309 residues of nucleoprotein are involved in the binding of the drugs with nucleoprotein.

There is a hypothesis that increasing concentration of nucleoprotein is a switch for replication and transcription of the genome. Due to this reason there is a need to find a drug effective against conserved regions of the influenza virus proteins involved in replication process such as nucleoprotein. The outcome of the current study will be helpful to identify the universal drug against all strains of influenza virus; to find out conserved residues within and among influenza virus nucleoprotein as a target for drug discovery [24].

## Conclusion

Due to strain variations among influenza virus, it is the need of the time to identify the conserved residues among different strains as a target for universal drug discovery based on compounds extracted from natural sources like the neem leaves. In the present study, nucleoprotein was selected and screened against compounds extracted from neem leaves using *in-silico* screening and molecular docking simulation techniques. The compound Hyperoside from neem leaf extract along with drugs LGH, Naproxen, BMS-885838 and BMS-883559 showed best interactions with conserved residues of the nucleoprotein. So these compounds have been identified for holding great potential for utilization as a universal drug against influenza strains. These observations require further considerations for *in-vivo* and *in-vitro* validations.

## Abbreviations

BMS-883559: N-[4-chloranyl-5-[4-[[3-(2-methoxyphenyl)-5-methyl-1,2-oxazol-4-yl]carbonyl]piperazin-1-yl]-2-nitro-phenyl]thiophene-2-carboxamide
BMS-885838: N-[4-chloranyl-5-[4-[[3-(2-methoxyphenyl)-5-methyl-1,2-oxazol-4-yl]carbonyl]piperazin-1-yl]-2-nitro-phenyl]pyridine-2-carboxamide
BMS-885986: N-[4-chloranyl-5-[4-[[3-(2-methoxyphenyl)-5-methyl-1,2-oxazol-4-yl]carbonyl]piperazin-1-yl]-2-nitro-phenyl]furan-2-carboxamide
CBX: 1H-1,2,3-triazole-4-carboxamide
HA: Hemagglutinin
LGH: 4-(2-chloro-4-nitrophenyl)piperazin-1-yl][3-(2-methoxyphenyl)-5-methyl-1,2-oxazol-4-yl]methanone
M: Matrix
NA: Neuraminidase
Naproxen: (+)-(*S*)-2-(6-methoxynaphthalen-2-yl)-propanoic acid
NP: Nucleoprotein
OMM: [4-(2-chloro-4-nitrophenyl)piperazin-1-yl][3-(2-chloropyridin-3-yl)-5-methyl-1,2-oxazol-4-yl]methanone]
OMS: [4-(5-bromanyl-3-methyl-pyridin-2-yl)piperazin-1-yl]-[3-(2-chlorophenyl)-5-methyl-1,2-oxazol-4-yl]methanone
